# Supplementary material for: Nutrition Literacy among Adolescents and Its Association with Eating Habits and BMI in Tripoli, Lebanon
Source: Diseases. 2021 Mar 29;9(2):25. doi: 10.3390/diseases9020025 (PMC8103266; doi:10.3390/diseases9020025)
Supplement: Supplementary file 1 [file diseases-09-00025-s001.pdf]

diseases

MDPI

Box plot analysis

The variability of the NLAI subscales was analyzed. Below are the box plots for the NH, MA, HFM, FLN, and FG scores are presented. As the box plots illustrate the scores are not quite different between the two BMI categories with good variability. In the text the median and IQR are reported which reflects the variability in the data and the indication of the results is explained in the results section

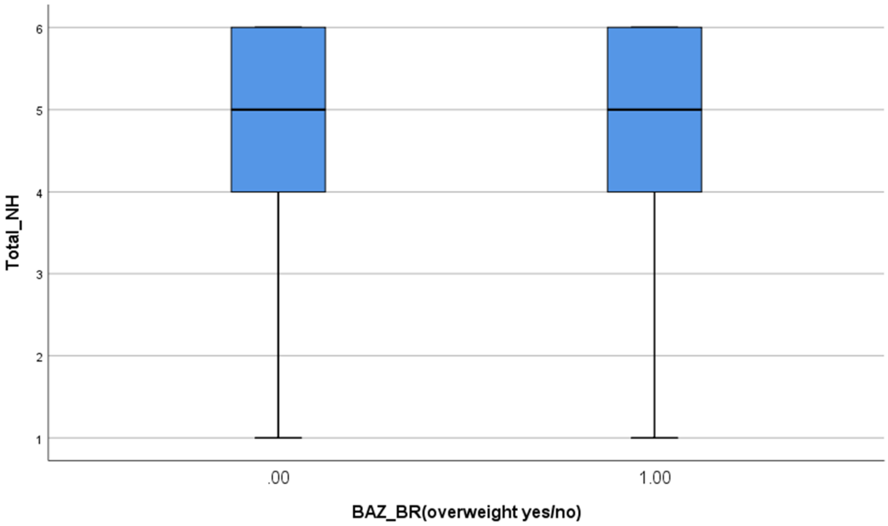

Figure S1. Box plot for total Nutrition and Health (NH)score by BAZ score category.

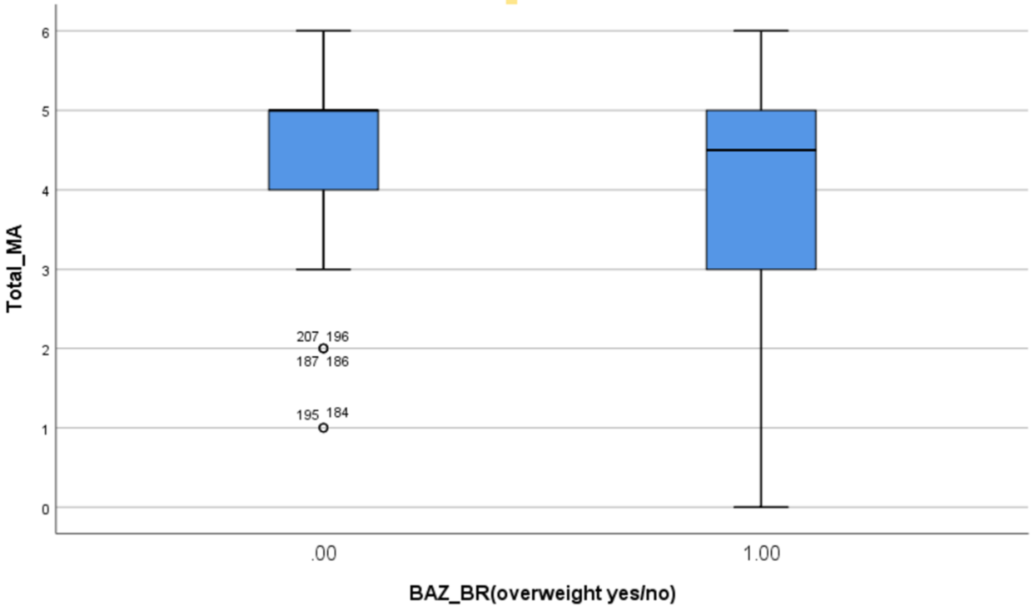

Figure S2. Box plot for total Macronutrient (MA) score by BAZ score category.

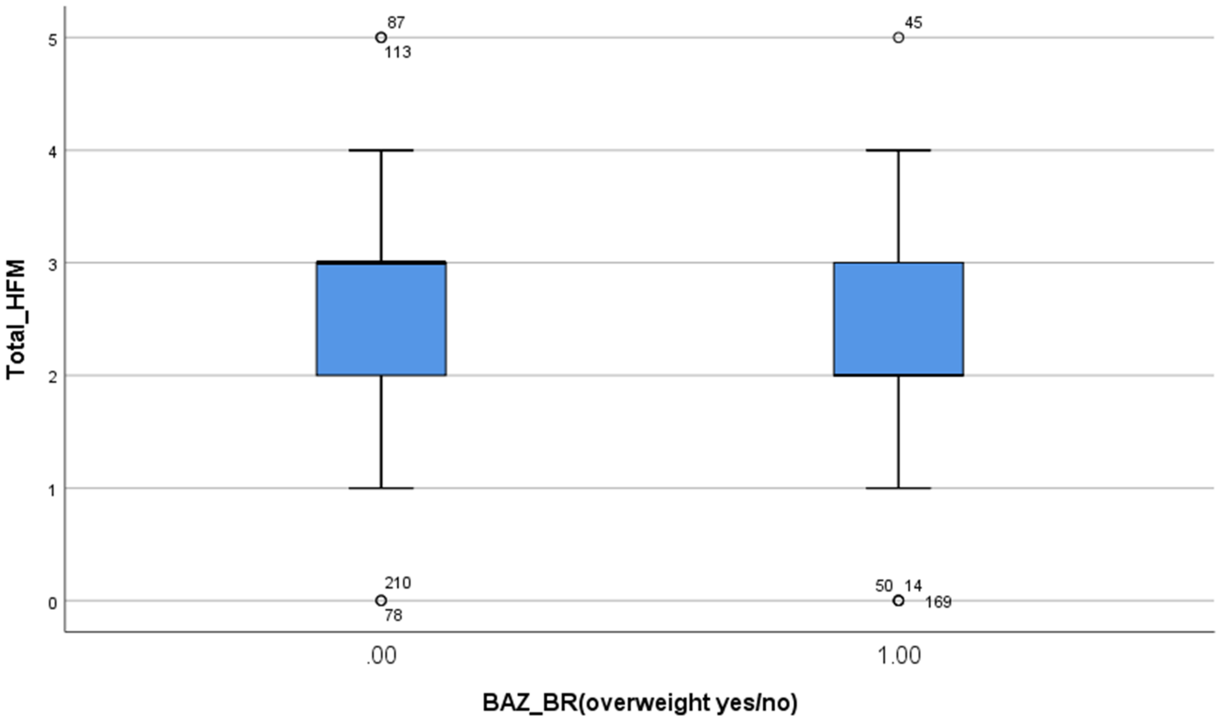

Figure S3. Box plot for total Household Food Measurement (HFM) score by BAZ score category.

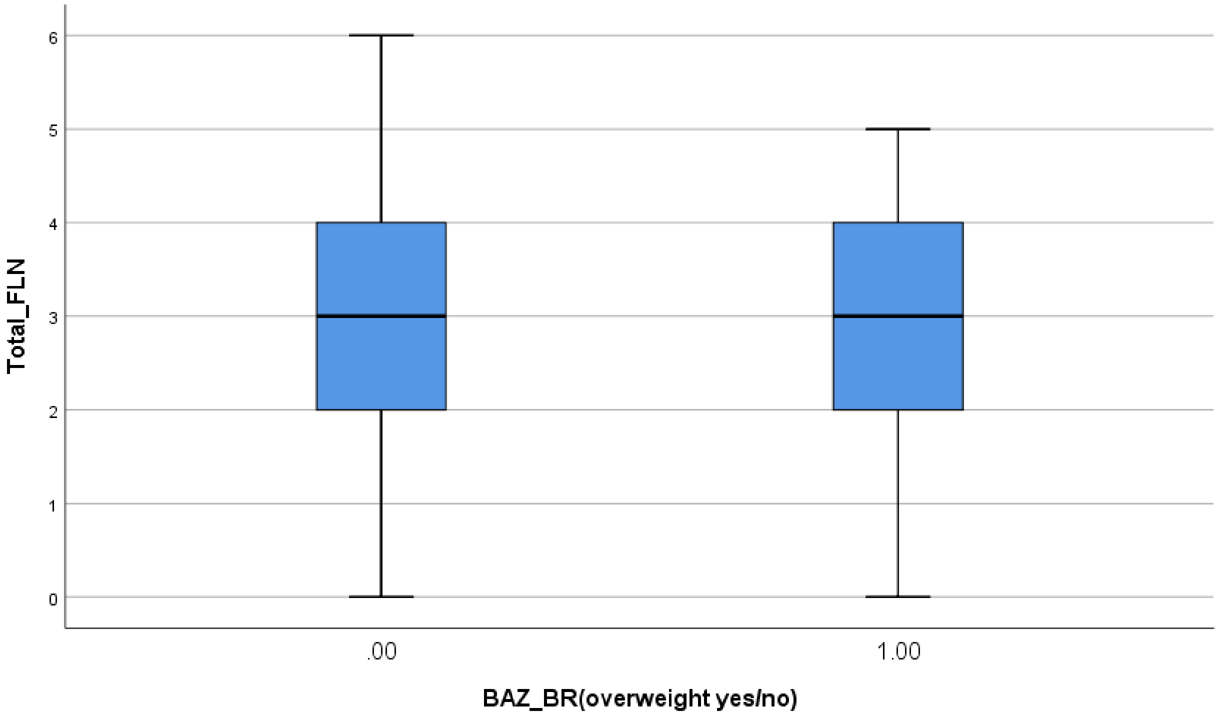

Figure S4. Box plot for total Food Label and Numeracy (FLN) score by BAZ score category.

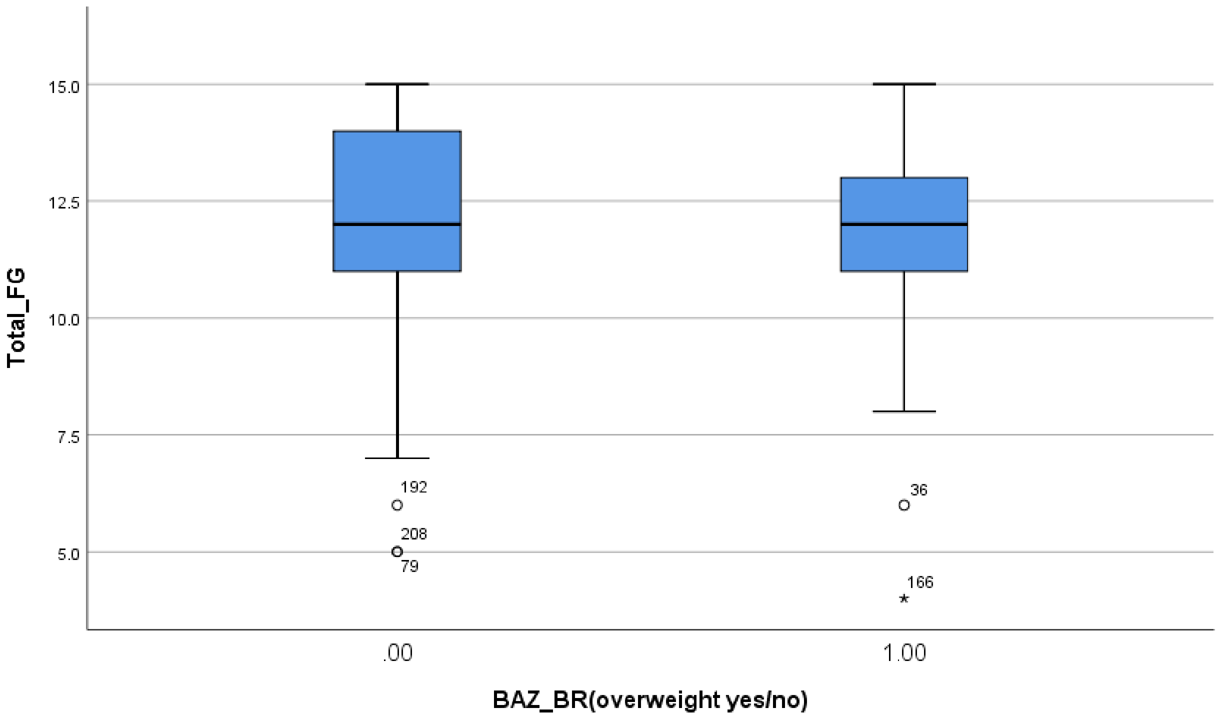

Figure S5. Box plot for total Food Group (FG) score by BAZ score category.

Reliability analysis

Cronbach's alpha was calculated for each nutrition literacy scale. A scale with less than 10 items tend to give low Cronbach's alpha. Loewenthal [1] and Cortina [2] have justified that the alpha coefficient can be lower if the scale had fewer than 10 items due to the profound effect a small number of items have on the alpha value (maximum number of items in this study is 6 items for each scale). Values for Cronbach's alpha are presented below for each scale and items.

Table S1. NH scale.

Item-Total Statistics					
	Scale Mean if Item Deleted	Scale Variance if Item Deleted	Corrected Item-Total Correlation	Squared Multiple Correlation	Cronbach's Alpha if Item Deleted
NH_1_S	3.7725	1.464	0.152	0.079	0.496
NH_2_S	3.8889	1.280	0.160	0.100	0.486
NH_3_S	3.9947	1.016	0.371	0.149	0.371
NH_4_S	4.1481	1.010	0.269	0.115	0.439
NH_5_S	3.9206	1.169	0.263	0.089	0.438
NH_6_S	4.0847	1.003	0.308	0.111	0.410
**Total scale Cronbach's alpha**					**0.491**

Table S2. MA scale.

Item-Total Statistics					
	Scale Mean if Item Deleted	Scale Variance if Item Deleted	Corrected Item-Total Correlation	Squared Multiple Correlation	Cronbach's Alpha if Item Deleted
MA_1_S	3.4709	1.304	0.185	0.083	0.402
MA_2_S	3.4868	1.219	0.280	0.119	0.356
MA_3_S	3.6878	1.067	0.246	0.092	0.363
MA_4_S	3.4392	1.269	0.330	0.150	0.353
MA_5_S	3.8413	1.092	0.172	0.053	0.420
MA_6_S	3.9259	1.112	0.154	0.058	0.432
**Total scale Cronbach's alpha**					**0.431**

Table S3. HFM scale.

Item-Total Statistics					
	Scale Mean if Item Deleted	Scale Variance if Item Deleted	Corrected Item-Total Correlation	Squared Multiple Correlation	Cronbach's Alpha if Item Deleted
HFM_1_S	2.3915	1.250	0.103	0.041	0.073
HFM_2_S	2.5503	1.121	0.178	0.168	-0.013[a]
HFM_3_S	2.6032	1.187	0.108	0.045	0.062
HFM_4_S	2.8730	1.760	-0.341	0.247	0.400
HFM_5_S	2.5291	1.102	0.203	0.256	-0.038[a]
HFM_6_S	2.6085	1.186	0.109	0.098	0.061
**Total scale Cronbach's alpha**					**0.136**

Table S4. FLN scale.

Item-Total Statistics					
	Scale Mean if Item Deleted	Scale Variance if Item Deleted	Corrected Item-Total Correlation	Squared Multiple Correlation	Cronbach's Alpha if Item Deleted
FLN_1_S	2.2011	1.247	0.070	0.043	0.313
FLN_2_S	1.9259	1.048	0.216	0.066	0.181
FLN_3_S	2.0476	1.301	-0.027	0.008	0.403
FLN_4_S	1.9630	.951	0.322	0.144	0.067
FLN_5_S	1.7672	1.158	0.154	0.075	0.244
**Total scale Cronbach's alpha**					**0.299**

Table S5. FG scale.

Item-Total Statistics					
	Scale Mean if Item Deleted	Scale Variance if Item Deleted	Corrected Item-Total Correlation	Squared Multiple Correlation	Cronbach's Alpha if Item Deleted
FG_GRAINS_S	3.1799	1.382	0.313	0.143	0.411
FG_VEGE_S	2.9259	1.548	0.235	0.078	0.456
FG_FRUIT_S	2.9206	1.520	0.266	0.132	0.440
FG_MPFB_S	2.9577	1.626	0.142	0.022	0.505
FG_DAIRY_S	2.8677	1.488	0.353	0.165	0.398
FG_FO_S	3.4550	1.622	0.209	0.098	0.468
**Total scale Cronbach's alpha**					**0.493**

References

1. Loewenthal K. *An introduction to psychological tests and scales.* 2nd ed.; Psychology Press: Philadelphia, PA, USA, 2001.
2. Cortina, J.M. What is coefficient alpha? An examination of theory and applications. *J. Appl. Psychol.* **1993**, *78*, 98–104, doi:10.1037/0021-9010.78.1.98.
